# ‘They treat you like their own’ Perspectives and Lived Experiences of Postpartum Women Seeking Health Services from Traditional Birth Attendants (TBAs) in Mayuge District East Central Uganda

**DOI:** 10.21203/rs.3.rs-7336278/v1

**Published:** 2025-10-08

**Authors:** Enid Kawala Kagoya, Proscovia Auma, Joshua Mugabi, Elizabeth Kawala, Deogratias Asabawebwa, Richard Mugahi, Brenda Doreen Mutunda, Andrew Twineamatsiko, Mercy Jackline Kabene, Kayemba Frank, Paul Waako, Kenneth Mugabe, Jackline Akello

**Affiliations:** Busitema University; Busitema University; Busitema University; Mayuge District Local Government; Mayuge District Local Government; Ministry of Health; Busitema University; Busitema University; Busitema University; Busitema University; Busitema University; Busitema University; Makerere University

**Keywords:** Traditional Birth Attendants, Postpartum Women, Maternal Health, Uganda, Qualitative Study, Health-Seeking Behavior, Cultural Beliefs, Health System Barriers, Community Influence

## Abstract

**Background:**

In Uganda, despite ongoing efforts to promote skilled birth attendance, many women in rural communities continue to deliver with Traditional Birth Attendants (TBAs). Understanding the reasons behind this preference and the nature of care provided by TBAs is critical for informing maternal health policy and community-level interventions.

**Objective:**

To explore the experiences, perceptions, and underlying factors influencing the continued use of TBAs for childbirth among postpartum women in Mayuge District, Eastern Uganda.

**Methods:**

A descriptive qualitative study was conducted in Mayuge District. In-depth interviews were held with 12 postpartum women who had delivered with TBAs within the past six months. Participants were purposively selected to capture variation in age, parity, and location. Data were collected using a semi-structured interview guide, audio-recorded, transcribed verbatim, and translated into English. Thematic analysis was conducted using both inductive and deductive coding in NVivo 13.

**Results:**

Five overarching themes emerged: (1) Health System Barriers, including poor access to health facilities, transport challenges, and mistreatment by health workers; (2) Perceived Benefits of TBA Care, such as affordability, emotional support, privacy, and flexible payment options; (3) Traditional Practices and Beliefs, with participants emphasizing cultural alignment, spiritual care, and birth rituals provided by TBAs; (4) Health Risks and Limits of TBA Care, reflecting awareness of complications, delayed referrals, and lack of emergency support; and (5) Community Influence, where social norms, peer recommendations, and collective trust in TBAs shaped decision-making. A cross-cutting theme of husband and family involvement also significantly influenced birth location choices.

**Conclusion:**

The continued reliance on TBAs is shaped by a complex interplay of structural barriers, cultural values, perceived advantages, and community dynamics. Interventions to improve maternal health in rural Uganda should address health system gaps, promote respectful and culturally sensitive facility-based care, and engage families and communities in birth preparedness efforts.

## Introduction

Globally, maternal and neonatal health remain pressing public health concerns, particularly in low- and middle-income countries (LMICs) where access to skilled birth attendance is limited [1]. According to the World Health Organization (WHO), approximately 295,000 women died in 2017 from preventable causes related to pregnancy and childbirth, with Sub-Saharan Africa accounting for roughly two-thirds of these deaths [2]. Uganda, like other countries in Sub-Saharan Africa, continues to face high maternal and perinatal mortality despite the implementation of numerous strategies aimed at promoting skilled birth attendance and facility-based deliveries [3].

The Ugandan government has expanded access to health facilities and trained health professionals, but significant disparities persist, particularly in rural areas [4]. Barriers such as long distances to health facilities, transportation challenges, poor provider attitudes, stock-outs of essential medicines and supplies, and deeply entrenched cultural practices often limit women’s utilization of formal health services [5]. In these contexts, Traditional Birth Attendants (TBAs) remain a prominent source of maternal care.

TBAs are often deeply embedded within communities, offering not only birth assistance but also emotional, social, and cultural support to women during pregnancy and childbirth [6]. National policy reforms have discouraged TBA-assisted deliveries in favor of skilled care; however, many women continue to seek TBA services due to trust, accessibility, affordability, and perceived cultural competence [7]. This presents a complex challenge for policymakers and health systems, who strive to improve maternal outcomes while respecting community preferences and indigenous health practices.

Previous studies have documented the continued reliance on TBAs in Uganda and similar settings, often highlighting their role in providing culturally appropriate care and filling systemic gaps left by formal health systems [8]. However, there is a need for qualitative research that directly captures the perspectives of women who recently sought care from TBAs. Understanding their motivations, experiences, and the sociocultural factors influencing their choices is essential for designing maternal health interventions that are context-sensitive and responsive to women’s lived realities [7]. This study was undertaken to explore the perspectives and experiences of postpartum women who delivered under the care of TBAs in a rural district in Eastern Uganda. Specifically, we sought to understand the factors that influence the preference for TBAs over skilled birth attendants, the nature of care provided by TBAs, and the broader sociocultural, economic, and systemic dynamics that shape maternal health-seeking behavior. By amplifying women’s voices, this study aims to contribute to the ongoing discourse on how best to integrate community-based actors into maternal health strategies while safeguarding maternal and neonatal outcomes

### Objective

To explore the experiences, perceptions, and underlying factors influencing the continued use of Traditional Birth Attendants (TBAs) for childbirth among postpartum women in a rural district in Eastern Uganda.

## Methods

### Study Design

This study employed a qualitative descriptive design to explore community perspectives and experiences regarding the use of Traditional Birth Attendants (TBAs) in maternal healthcare. A qualitative approach was appropriate to elicit rich, in-depth insights into perceptions, beliefs, and decision-making processes surrounding childbirth care in a rural Ugandan context.

### Study Setting and Population

The study was conducted in Mayuge, a rural district in Eastern Uganda characterized by limited access to formal maternal health services. The study population comprised postpartum women who had delivered within the previous 12 months and had utilized the services of TBAs during their most recent pregnancy or delivery.

### Sampling and Recruitment

Participants were selected using purposive sampling, achieving maximum variation in age, parity, and geographic location within the district. Local health workers and community leaders assisted in identifying eligible participants. Recruitment continued until data saturation was reached, defined as the point at which no new themes were emerging from subsequent interviews.

### Data Collection Methods and Tools

Data were collected through in-depth interviews (IDIs) using a semi-structured interview guide developed in English and translated into the local language. The guide included open-ended questions and probes covering themes such as maternal care experiences, perceptions of TBAs and health workers, cultural practices, and barriers to facility-based care.

Interviews were conducted in a private setting by trained qualitative researchers fluent in the local language. Each session lasted approximately 45–60 minutes and was audio-recorded with participants’ consent. Field notes were taken to supplement the recordings and capture non-verbal cues and contextual information.

### Data Management and Transcription

Audio recordings were transcribed verbatim and translated into English. Transcripts were reviewed alongside audio files to ensure accuracy. All transcripts were anonymized, with identifiers removed prior to analysis. Data were stored in password-protected digital files accessible only to the research team.

#### Data Analysis Plan:

We used thematic analysis, following the six steps outlined by Braun and Clarke (2006): (1) familiarization with the data, (2) generation of initial codes, (3) searching for themes, (4) reviewing themes, (5) defining and naming themes, and (6) producing the report.

### Data Coding Approach

Initial coding was conducted manually and later refined using NVivo 13 software. A combination of deductive coding based on the interview guide and inductive coding emerging directly from the data was employed. Two independent coders read all transcripts to identify initial codes. Discrepancies were discussed and resolved through consensus, and a final codebook was developed.

Codes were grouped into subthemes and then organized under five overarching themes based on conceptual similarity and frequency across participants. Code saturation was achieved when no new codes emerged in the final interviews.

### Data Appraisal and Trustworthiness

To ensure trustworthiness, we employed the criteria of credibility, dependability, confirmability, and transferability as outlined by Lincoln and Guba. Credibility was enhanced through peer debriefing and member checking with a subset of participants. Dependability was supported by maintaining an audit trail of coding decisions. Confirmability was ensured by reflexive journaling and independent coding by two researchers. Transferability was addressed by providing thick descriptions of the study context and participant narratives.

### Data Presentation

Findings were presented thematically, with the most illustrative verbatim quotes used to support each theme and subtheme. Pseudonyms and generic identifiers were used to maintain anonymity. Themes were organized to reflect the participants’ lived experiences and to provide a holistic understanding of the community’s use of TBAs in maternal care.

### Ethical Consideration

The study adhered to the Belmont Report principles of respect for persons, beneficence, and justice. Ethical approval was sought and obtained from the Research Ethics Committees (REC) of Mbale Regional Referral Hospital No.MRRH-2023–342. Written informed consent was obtained from all participants before data collection. Interviews were conducted in private locations to ensure confidentiality and minimize social risk. No personal identifiers were retained in the final data. Audio files and transcripts were encrypted and securely stored.

## Results

Twelve postpartum women, aged 21 to 38 years, participated in the study, all of whom resided in either rural or peri-urban areas of Eastern Uganda (see Table 1).

### Thematic Areas

A total of five overarching themes emerged from the analysis, reflecting participants’ experiences and perceptions regarding the use of Traditional Birth Attendants (TBAs) in maternal care. These included: (1) health system barriers; (2) perceived benefits of TBA care; (3) traditional practices and beliefs; (4) health risks and limits of TBA care; and (5) community influence. A cross-cutting domain, husband and family involvement, also played an important role in shaping decisions about birth care (see Table 2).

### Theme One: Health System Barriers

Participants consistently described multiple barriers within the formal health system that hindered their ability to access timely and respectful maternity care. These systemic challenges not only discouraged women from utilizing health facilities but also reinforced their reliance on TBAs, who were perceived as more accessible and responsive to their needs. The barriers included financial constraints, negative provider attitudes, delays in care provision, and corruption, all of which cumulatively contributed to poor patient experiences and decreased trust in formal healthcare services.

### Subtheme One: Cost of Services and Supplies

A recurring challenge for many women was the financial burden associated with facility-based delivery. Despite policies advocating for free maternity services, participants reported being required to purchase essential delivery materials before care could be provided. This included items such as gloves, cotton wool, disinfectants, and delivery kits. Several women narrated experiences where they were refused attention or delayed care until they produced these supplies. This requirement disproportionately affected poorer women, leading to delays in receiving skilled care or complete avoidance of the formal system altogether.

“She said...I won’t touch on her...until you have gotten those things.” (NEBU, 37 years, Buwaliima)

“She said for her she touches only people with beddings, a razor blade, and a thread and sometimes we don’t have them.” (NEBU, 37 years, Buwaliima).

“They are selling everything to us.” (NPMY, 25 years, Mayuge Town Council)

This financial barrier effectively excluded vulnerable women and undermined the government’s policy of free maternal health services.

### Subtheme Two: Disrespect and Neglect from Health Workers

Disrespectful treatment by health workers emerged as a significant deterrent to facility use. Participants frequently described feeling ignored, neglected, or mistreated during labor and delivery. They recounted incidents where they were bypassed by healthcare staff, left unattended for long periods, or spoken to harshly. This perceived lack of empathy and care contributed to feelings of humiliation and mistrust toward the formal health system. The emotional toll of such experiences fostered a reluctance to return to health facilities for future deliveries.

“They just bypassed us...until we went back home without gaining anything.” (NOKA, 31 years, Kasutayime)

“There is a midwife who only wants us to put on white clothes like white nicker, bra etc, if you don’t have them, she won’t deliver you.” (KJBU, 26 years, Buguwa)

Such experiences of disrespect discouraged women from seeking skilled birth attendance and reinforced the perception that TBAs offered more compassionate and culturally sensitive care.

### Subtheme Three: Delay in service delivery

Participants reported prolonged waiting times, even for women in active labor, as a common occurrence in public health facilities. Delays were attributed to staff shortages, overwhelming patient loads, and inefficiencies in the system. These delays often resulted in women becoming discouraged, exhausted, or forced to return home without receiving care. The frustration of waiting for many hours without attention exacerbated physical distress and fear during labor, pushing women to seek alternatives outside the formal health system.

“We would sit from morning to like 5pm, only one midwife around, ohh God...she shakes her head...” (NSLW, 38 years, Lwandela)

“Some of us come as far as Mpungwe but those nurses keep you waiting until late, you end up spending a night on a veranda because of fear of delivering while on the way back which these TBAs don’t do, you come and they treat you there and then.” (KFBU, 21 years, Bukatuube)

Such delays compromised timely access to skilled care and increased the likelihood of adverse birth outcomes.

### Subtheme Four: Informal Fees and Corruption

Informal payments and corruption within health facilities were frequently cited as major obstacles to care. Despite official policies of free maternal services, many participants described being solicited for “appreciation money” or bribes, sometimes even after the delivery had occurred. This practice not only imposed an additional financial burden but also deepened distrust in the formal health system. Participants expressed frustration that health workers demanded money regardless of the quality or timeliness of care provided.

“She asked for appreciation money...yet she found the baby was out, I wondered why she needed the money yet she earns a salary” (KRKi,29 years,Kigulu)

“You first talk to a VHT, then you go to a nurse, then the nurse sends you to a doctor, why all that yet at every point you leave some ka money, yet these TBAs, that process is not there.” (NABK,34 years, Bukabooli)

Such corrupt practices eroded confidence in public health services and reinforced women’s preference for TBAs, who were perceived as more honest and approachable.

### Theme Two: Perceived Benefits of TBA Care

While participants acknowledged certain risks associated with TBA care, many emphasized the compelling advantages that drew them to seek support from TBAs instead of formal health facilities. These perceived benefits included affordability, emotional and physical support, responsiveness, and continuity of care. For many women, TBAs provided a dignified, humanized, and culturally familiar approach to childbirth that stood in sharp contrast to their experiences within the formal health system. This theme highlights how positive perceptions of TBAs played a central role in shaping care-seeking behavior.

### Subtheme One: Affordability

Affordability was a key driver of preference for TBA care. Participants explained that TBAs offered a more affordable option, especially for women who were constrained by the indirect costs associated with facility deliveries, such as transport, medical supplies, and informal fees. In contrast to health facilities that often demanded upfront purchase of delivery items, TBAs provided comprehensive care at a fixed and often negotiable cost.

“I paid 15,000 UGX...including antenatal medicines which is cheaper than when I go to a hospital they start asking for things which I can’t afford.” (NMRW, 38 years, Waitambogwe)

“TBAs take whatever money you have, they don’t have fixed prices compared to some health facilities where delivery has a fixed amount, without it, no service.” (NSLW, 38 Years, Lwandela)

This relatively low cost provided significant relief for women with limited resources and reduced the financial stress associated with childbirth, making TBAs an appealing and realistic option for many.

### Subtheme Two: Compassion and Emotional Support

Participants emphasized the compassion and emotional support offered by TBAs. Unlike the often impersonal and neglectful treatment reported in formal settings, TBAs were described as warm, nurturing, and attentive. Their support extended beyond clinical care to include emotional reassurance and physical comfort during labor. Several women shared that TBAs not only assisted with childbirth but also prepared meals, offered encouragement, and stayed present throughout the process.

“She cooks you some food and motivates you until you are left to go home, if you come alone, her girls here can help you wash etc.” (NMBW, 27 years, Buwaiswa)

“There is always a kettle on fire at their place, you take tea, they give you food and sometimes even wash for you’re the blood after delivery which does not happen in our hospitals.” (KRKI, 29years, Kigulu)

“There is a TBA in Buwaiswa, he has a radio in the delivery room, you deliver with some nice music on, then after delivery, calls his wife to serve you something to eat and only discharges you when you are well.” (NMBW, 27Years, Buwaiswa)

Such care fostered a deep sense of trust, safety, and emotional wellbeing, elements that participants found lacking in formal health facilities.

### Subtheme Three: Responsiveness and Personalized Care

The responsiveness of TBAs was a defining feature that distinguished them from health facility staff. Participants appreciated the immediate attention and individualized care they received from TBAs, particularly during labor. Unlike the delays and long queues experienced in public health facilities, TBAs were described as available at all hours and willing to respond quickly to a woman’s needs. This level of attentiveness contributed to a perception that TBAs genuinely cared about their clients and prioritized their wellbeing.

“They work on us faster because they care more about us.” (NKMKI, 23 Years, Kikuube))

“These TBAs know our problems so when you come they will treat you like their own, provide all the necessary care without hesitance, even when you don’t have money, just bring your hen, give her and everything will be sorted there and then.” (NMRW, 38 years, Waitambogwe)

This perceived dedication and timeliness reinforced the view that TBAs were more dependable, especially in urgent or emotionally vulnerable moments.

### Subtheme Four: Continuity of Care

Continuity of care after delivery emerged as another valuable aspect of TBA services. Unlike health facilities, where mothers were often discharged shortly after delivery, TBAs were seen as offering extended support and recovery time. Women expressed comfort in being allowed to rest and recover under the watchful eye of someone familiar and caring. The option to remain in the care setting after childbirth was described as reassuring, especially for first-time mothers or those with complications.

“You even sleep there...she cannot discharge you at that moment.” (KPMP,Female,28 years, Mpungwe)

### What do you mean?

“She will leave you in her room, monitor you until when you are fine that’s when she will let you go.” (KPMP, Female,28 years, Mpungwe)

“I went to deliver my second baby from Nalukidis place, after delivery, she kept coming to my home to check on me and my baby, teach me some practices like cord leaning etc which health workers don’t do.” (NOKA,Female,31 years, Kasutayime)

This approach not only provided physical recuperation but also created a sense of emotional security and maternal bonding that was often missing in formal healthcare encounters.

### Theme Three: Traditional Practices and Beliefs

Participants placed considerable value on the way Traditional Birth Attendants (TBAs) incorporated culturally familiar practices, herbal remedies, and spiritual interpretations into maternal care. These practices reinforced the perception that TBAs offered holistic care aligned with both physiological and symbolic aspects of childbirth. TBAs were not only viewed as skilled in birth assistance but also as custodians of indigenous knowledge capable of addressing both medical and spiritual concerns.

### Subtheme One: Use of Herbal Medicine

The use of herbal preparations was a common feature of care provided by TBAs. Participants described these remedies as both preventive and curative, used to ease labor pains, safeguard the pregnancy, and promote overall well-being. These herbs were often administered as drinks or ointments to be applied externally.

“She gave me...to smear on the belly and...to drink.......after drinking the herbs, I felt like pushing the baby, she put me in the delivery room, pushed my baby boy and left the next day.” (NABK, 34 years, Bukabooli)

“There are also these potato leaves that they give you to stop vomiting in the early stages of pregnancy, they helped me a lot because whenever I would vomit a lot, I would end up lacking energy and feel week.” (NKMKI, 23 Years, Kikuube)

Such practices were deeply embedded in local traditions and were seen as safe, effective, and part of a continuum of ancestral knowledge that supported maternal health.

### Subtheme Two: Pregnancy Management Rituals

TBAs were sought for their expertise in culturally specific pregnancy conditions, such as *Nabuguma* (a condition characterized by unexplained pain or swelling), which were not readily addressed in biomedical settings. Participants described timed rituals and consultative visits guided by local belief systems to manage pregnancy and avert complications.

“You calculate...then the months pass...you say I will go to Mrs. Bizibu., she is one of our best traditional Birth Attendants her, she will tell you all what you need to do even if you are not term like taking some herbs, smoking at night etc. Smoking was normally to aid movement of the baby and the herbs were basically for restoration of energy, opening the cervix etc.” (KPMP, 28 years, Mpungwe)

“My first pregnancy, I had “Deeni”(a postdate pregnancy), I kept going to her every morning and evening to get some “Nfwoodo” and did some cultural rituals in her house, in just one week, I gave birth.” (NEBU, 37 years, Buwalima)

These rituals enhanced the sense of control and spiritual preparedness for childbirth, reinforcing TBAs’ roles as trusted maternal caretakers.

### Subtheme Three: Spiritual or Symbolic Interpretations

Many TBAs were believed to possess the ability to interpret the fetus’s position, maternal health status, or labor progression through a spiritual or symbolic lens. This spiritual insight was highly valued by participants, particularly when conventional diagnostics were inaccessible or perceived as indifferent.

“She touches the belly...tells you the baby is lying transversely and if the baby is not positioned well, she will give you treatment to change the babies position especially when you are remaining with a few days to give birth, this saved me from C-section.” (NMRW,, 38 years, Waitambogwe)

These interpretations added a spiritual reassurance that complemented physical care, making the birth experience more meaningful and affirming for mothers.

### Theme Four: Health Risks and Limits of TBA Care

Despite their trust in TBAs, participants openly acknowledged the limitations of TBA care, especially during complications. This theme illustrates the complex balancing act women performed between cultural preference and recognition of biomedical necessity.

### Subtheme One: Complex or Emergency Cases

Participants shared stories of TBAs encountering complications such as hemorrhage, prolonged labor, or neonatal distress. In such cases, TBAs often relied on informal or ad hoc responses, including calling friends with some health training.

“She called her friend...a health worker...put the cannula.” (NABK, 34 years, Bukabooli))These practices reflected resourcefulness but also highlighted a lack of preparedness and the absence of standardized emergency protocols.

### Subtheme Two: Informal Referral Systems

Rather than using formal referral channels, TBAs often depended on social or personal networks to escalate cases beyond their capacity. This informal system, while sometimes effective, delayed access to critical care and underscored the structural disconnect between TBAs and the formal health sector.

“She said she had a friend who was operating a private clinic ...called her and she came with a drip but they failed to run it well but I later moved to health facility.” (NABK, 34 Years, Bukabooli)

They sometimes invite their fellow TBAs to support especially for conditions like Deeni (Post date), Makiro (preeclampsia)etc and if it fails, they rush you on a bodaboda (motorcycle) to the hospital.” (NMBW, 27Years, Buwaiswa)

Such accounts raised concerns about delayed interventions and the absence of timely coordinated emergency response.

### Subtheme Three: Lack of Formal Procedures

Participants described instances in which TBAs were unprepared to manage complications, particularly those involving the newborn. These cases were marked by confusion and improvised solutions that sometimes failed to avert harm.

“She really struggled when I gave birth to my baby, I don’t what happened but the baby stopped breathing, we panicked, it was at night......she didn’t know what to do.

### So what happened?

She brought that thing that holds a papaw leaf, put it in the baby’s anus, started plowing air in the anus nonstop, tried it several times until the bay started breathing

### Do you think it was a good method?

Yes, because at that time, I needed by baby

### So how is that bay now?

The baby died before it made 3 months.... She cries....” (KPMP, 28 years,Mpungwe)

These limitations were acknowledged even by those who otherwise preferred TBA care, reinforcing the need for better linkages with biomedical providers during emergencies.

### Theme Five: Community Influence

Community narratives, norms, and social networks played a decisive role in shaping women’s choices around childbirth. The influence of peers, family, and village elders along with shared stories of past experiences, helped to legitimize TBA care as an acceptable and even preferable option.

### Subtheme One: Peer Influence and Word of Mouth

Participants often cited the experiences of neighbors, friends, and relatives in shaping their care-seeking decisions. Stories of neglect, abuse, or delays at government facilities were widely circulated and influenced expectations of care.

“The women in the village...told me that they had gone to the government hospitals, but they weren’t helping, I also gave it a try and trust me, these people are good” (NMBW, 27Years, Buwaiswa)

These narratives built collective distrust toward formal health systems while simultaneously validating TBA care through social endorsement.

### Subtheme Two: Trust in TBAs

TBAs were often regarded as community figures with reputations built on years of compassionate and effective care. Participants frequently emphasized the trust TBAs had earned within their communities, citing their kindness, accessibility, and willingness to serve without judgment.

“I like them very much...because of their ways, they care about the patients, and they are not strict on what to bring and what not to bring.” (NSLW, 38 years, Lwandela)

“One time I went there just to find out how much it was to deliver from her place, she instead gave me some medicines that helped me upto when I reached time for delivering and went there, she delivered me and told me to pay Ugshs 20,000 which I even paid like after a month because I didn’t have money.” (NOKA, 31 years, Kasutayime)

This deep-seated trust functioned as social capital that TBAs leveraged to sustain their practice even in areas with access to formal facilities.

### Cross-Cutting Theme: Husband and Family Involvement

Although not always explicitly emphasized, husbands and family members were often central to decision-making regarding where and with whom a woman would give birth. In many cases, the decision to seek TBA care was supported or initiated by husbands, especially when prior facility-based births were perceived as traumatic or disappointing.

“He accepted because of the condition of the first pregnancy, we lost our first pregnancy because we went to Buwaiswa, the Bawawo (health workers) left us there or a full day, we went back the next day, by the time I delivered, I was told the bay was tired and it died, my husband told me never to go back to government hospitals.” (NMBW, 27years, Buwaiswa)

“I went to a TBA for ANC, my husband realized it was not costly in terms of transport, and even the time spent there, he kept escorting me to there and even took me there to deliver, and my babies are here, all delivered by a TBA.” (KJBU, 26 years, Buguwa)

These findings point to the importance of engaging men and broader family networks in maternal health dialogues to support informed and safe choices across care settings.

## Discussion

This study offers critical insights into the complex factors influencing maternal care-seeking behaviours in Mayuge district, East Central Uganda, particularly women’s continued preference for Traditional Birth Attendants (TBAs) despite the availability of formal maternal health services. The findings reveal deeply embedded structural, socio-cultural, economic, and interpersonal dynamics shaping women’s decisions about where and with whom to give birth. When examined in a broader context, these patterns resonate with studies conducted in other low- and middle-income countries, suggesting that while local nuances are unique, the barriers and motivations influencing maternal care utilization are often similar across settings [9].

A key barrier identified in this study was the financial burden women face in accessing facility-based maternity care. Despite Uganda’s policy of free maternal health services, participants reported having to purchase delivery items such as gloves, cotton wool, disinfectants, and delivery kits as a prerequisite for receiving care. This contradiction between policy and practice is not unique to Uganda. Similar discrepancies have been noted in Nigeria, where women reported being charged for basic supplies and medications even in public hospitals [10]. In Tanzania, a study found that women were frequently required to pay for services and supplies despite official mandates for free services [11]. These hidden costs deter poor and marginalized women from accessing skilled care, resulting in either delayed health-seeking or avoidance of formal care altogether.

This paradox between promised free services and out-of-pocket expenses effectively undermines equity in maternal healthcare. It suggests that policy implementation gaps and weak regulatory mechanisms continue to marginalize vulnerable groups. The findings support broader arguments for increased accountability and stronger health financing systems to ensure that public health services are not only theoretically free but also practically accessible and equitable [12].

Disrespect and abuse by health providers was another critical deterrent. Women in this study shared painful experiences of being ignored, harshly spoken to, and left unattended during labor experiences that severely undermined their trust in formal healthcare providers. This aligns with the work of [13], whose systematic review across 34 countries found widespread mistreatment of women during childbirth, including verbal abuse, abandonment, and non-consented care. Similarly, in Kenya, [14] documented how disrespectful care discouraged women from delivering at health facilities, fueling distrust and driving them toward informal providers like TBAs.

In Ghana, [15] found that women, especially adolescents, feared mistreatment by midwives and often opted for home delivery to preserve their dignity. These shared experiences reflect systemic issues of provider burnout, poor supervision, lack of accountability, and inadequate training in respectful maternity care. They underscore the urgent need for health system reforms that prioritize client-centered care and uphold women’s rights during childbirth.

Delays in receiving attention at health facilities, even during active labor, were also widely reported. These were attributed to staff shortages, long queues, and inefficient triage systems. Such delays not only increased anxiety and physical suffering but also resulted in some women being turned away or delivering outside the facility. These findings echo those from [16] found that long wait times and overcrowding in public hospitals often forced women to return home without care. In Malawi, staff shortages led to significant delays in the administration of life-saving obstetric interventions [17].

The inefficiency compromises the principle of timely access to skilled care a key determinant of maternal and neonatal survival[16]. When health systems fail to deliver prompt services, women may rationally opt for TBAs, who are readily available and willing to respond quickly [18]. These findings suggest a need for targeted investments in human resources, facility infrastructure, and patient flow systems to enhance responsiveness.

Participants frequently cited informal payments and corruption as pervasive obstacles to care. These practices undermined trust in public health services and contradicted government commitments to provide free maternal care. Studies in Bangladesh have documented similar issues, where women were expected to pay unofficial fees to receive care or expedite services [7].

Women in this study consistently reported that TBAs offered a more affordable option, often accepting small payments, payment in kind (e.g., chickens), or installment arrangements. This flexibility contrasts with the rigid payment demands of formal facilities and reflects broader findings in Nepal and Bangladesh, where TBAs were seen as economically accommodating [19],[20].

In resource-constrained settings, affordability is often the most immediate determinant of access. Even when facility services are technically free, indirect costs such as transportation, food, and supplies can be prohibitive. TBAs meet women where they are, both geographically and financially, making them a vital part of the maternal health safety net for the rural poor.

The emotional support provided by TBAs emerged as a powerful motivator for their continued utilization. Participants described TBAs as caring, attentive, and emotionally present throughout labor and postpartum recovery. Similar findings have been reported in Ghana [19] where TBAs were viewed as mother figures who guided women through childbirth in nurturing and empowering ways.

In contrast, the impersonal nature of formal health services often left women feeling alienated and unsupported. This contrast reinforces the importance of integrating emotional and psychosocial care into facility-based maternity services. In [21]. Respectful care models that emphasize empathy, presence, and companionship have been shown to improve facility delivery rates.

TBAs were praised for their availability, individualized attention, and quick response to labor. Participants valued their ability to be seen immediately without the bureaucratic delays of hospitals. This immediacy was critical during emergencies or at night when transportation to facilities posed additional risks. In Pakistan [22] noted that women favored TBAs for similar reasons: proximity, rapid response, and familiarity.

This responsiveness also fostered a sense of personalised care. TBAs knew their clients personally and offered services in culturally comfortable environments. This level of intimacy and trust is rarely replicated in formal health facilities, where high patient loads and institutional detachment often hinder relational care.

Participants appreciated the extended postnatal care TBAs provided, including home visits and monitoring after delivery. This continuity of care is often absent in formal systems where mothers are discharged shortly after delivery with minimal follow-up. In Tanzania, researchers found that TBAs often assisted with breastfeeding, cord care, and postpartum hygiene for days or weeks after birth [11]. Such support is particularly crucial for first-time mothers and those recovering from difficult births. It also allows early detection of complications and provides an opportunity for reinforcing health education. Strengthening postnatal outreach by community health workers or involving trained TBAs could bridge this gap and reduce maternal and neonatal risks [23].

The integration of herbal medicine into TBA care reflects longstanding indigenous knowledge systems that continue to hold significance for many women. Similar practices have been documented in Ghana, where traditional remedies are used to ease labor, prevent miscarriage, and promote postpartum recovery [15]. While the efficacy of such treatments remains contested in biomedical circles, their symbolic and emotional value is unquestionable. For many women, herbal remedies provide a tangible connection to ancestral wisdom and a sense of cultural continuity. Recognizing and studying these practices could offer opportunities for safer integration or referral in maternal health programs [19].

Cultural rituals around pregnancy management, such as timed visits, spiritual protection, and symbolic interpretations of fetal position were important elements of TBA care. In Zimbabwe and Peru, similar rituals and beliefs about “spiritual blockages” or ancestral influences on labor have been documented [24].

These practices may serve to reduce anxiety, affirm women’s experiences, and increase their sense of control. Although not always aligned with biomedical logic, they fulfill critical emotional and cultural needs. Ignoring or dismissing them can further alienate women from formal services. Cultural competence training for formal providers and respectful accommodation of symbolic needs may help bridge this gap.

While TBAs are trusted and valued, their limitations, particularly in handling obstetric emergencies, were acknowledged by participants. Women recounted instances of hemorrhage, fetal distress, or birth asphyxia where TBAs were unprepared or resorted to improvised solutions. Similar concerns have been reported in Bangladesh, where TBAs lack training in managing complications or recognizing danger signs [20]. The reliance on informal referrals or delayed transfers to hospitals increases the risk of adverse outcomes. These findings call for strategic partnerships that do not aim to eliminate TBAs but to link them with the formal system through training, supervision, and referral networks.

Social and peer influence played a decisive role in legitimizing TBA care. Narratives of neglect, death, or disrespect in formal facilities were widely shared and often deterred women from seeking skilled care. These social endorsements mirror findings in Nigeria, where community narratives function as powerful vehicles for shaping health behavior [25]. This highlights the need for community-level interventions that address not only individual behaviors but also collective perceptions and norms. Engaging community leaders, elders, and satisfied mothers as maternal health champions may help shift social narratives toward safer practices.

Men and family members played a pivotal role in care-seeking decisions. In cases of prior trauma or poor experiences in hospitals, husbands actively encouraged to delivery with TBAs. This aligns with findings from low and middle-income countries where husbands, mothers-in-law, and other relatives exert considerable influence over maternal health decisions [26], [27]and [28]

Maternal health interventions that fail to engage men and family networks risk limited uptake and sustainability. Programs promoting respectful maternity care, emergency preparedness, and facility delivery should actively involve male partners and family members to enhance informed decision-making and support [6].

This study affirms that despite efforts to expand skilled birth attendance in Uganda, substantial barriers remain that discourage women from utilizing formal maternal health services. Women’s reliance on TBAs reflects both structural deficits in the health system and the enduring appeal of culturally resonant, affordable, and compassionate care. These findings call for a dual approach, strengthening facility-based care while recognizing, engaging, and partnering with TBAs. Cross-country evidence shows that integrated models that honor women’s preferences while improving safety and emergency response are not only feasible but essential for advancing maternal and newborn health equity

## Conclusion

This study highlights that Traditional Birth Attendants continue to play a significant role in childbirth care in rural Eastern Uganda due to their accessibility, culturally sensitive and respectful care, perceived competence, and deep community integration. Despite ongoing efforts to promote facility-based deliveries, many women prefer TBAs because of systemic barriers such as distance, poor treatment by health workers, and lack of resources in formal health facilities. These findings underscore the need for maternal health policies and interventions that go beyond simply promoting institutional delivery to also address the quality of care, respect, and cultural relevance in maternity services. Recognizing and engaging TBAs as partners within the maternal health continuum may offer an opportunity to improve birth outcomes while respecting women’s preferences and realities.

### Limitations

This study has several limitations. First, the qualitative design and purposive sampling limit the generalizability of the findings beyond the study area. Second, participants’ responses may have been influenced by social desirability bias, particularly regarding their perceptions of health facilities and TBAs. Third, the study did not include the perspectives of health workers, TBAs themselves, or male partners, which could provide a more comprehensive understanding of the factors influencing childbirth choices. Despite these limitations, the rich qualitative data provide valuable insights into the lived experiences of postpartum women in this context.

### Recommendations

Based on the findings, several recommendations emerge. Health policymakers and program implementers should prioritize improving the quality of care in health facilities, focusing on respectful maternity care and reducing mistreatment. Efforts to strengthen the health system by ensuring availability of skilled staff, essential supplies, and timely services are critical. Additionally, integrating TBAs into the formal health system through training, supervision, and defined referral roles could leverage their community trust and accessibility to improve maternal and newborn health outcomes. Finally, further research involving TBAs, health workers, and community members is needed to develop culturally appropriate strategies that bridge the gap between traditional and biomedical care

## Figures and Tables

**Table 1:** Social demographic characteristics of participants

Identifier	Age	Gender	Location
NPMY	25 years	Female	Mayuge town Council
NOKA	31 years	Female	Kasutayime
KPMP	28 years	Female	Mpungwe
NMBW	27 years	Female	Buwaiswa
NABK	34 years	Female	Bukabooli
NSLW	38 years	Female	Lwandela
KJBU	26 years	Female	Buguwa
NKMKI	23 years	Female	Kikuube
KFBU	21 years	Female	Bukatuube
KRKG	29 years	Female	Kigulu
NMRW	38 years	Female	Waitambogwe
NEBU	37 years	Female	Buwalima

**Table 2:** Summary of Thematic areas

Theme	Subtheme	Description	(Quotes)
Health System Barriers	Cost of services and supplies	Hospital requires purchase of delivery materials, which some mothers can’t afford	“She said...I won’t touch on her...until you have gotten those things.”
	Disrespect and neglect from health workers	Negative experiences with hospital staff, including being ignored or mistreated	“They just bypassed us...until we went back home without gaining anything.”
	Delay in service delivery	Long waiting times in government facilities	“We would sit from morning to like 5pm.”
	Informal fees and corruption	Health workers asking for money informally	“She asked for appreciation money...yet she found the baby was out.”
Perceived Benefits of TBA Care	Affordability	TBAs provide services at low or no cost	“I paid 15,000 UGX...including antenatal medicines.”
	Compassion and emotional support	TBAs offer comfort, food, and motivational support	“She cooks you some food and motivates you.”
	Responsiveness and personalized care	TBAs respond faster and more attentively to clients’ needs	“They work on us faster because they care more about us.”
	Continuity of care	TBAs allow mothers to stay post-delivery and provide follow-up support	“You even sleep there...she cannot discharge you at that moment.”
Traditional Practices and Beliefs	Use of herbal medicine	TBAs provide herbal treatments for pregnancy and labor	“She gave me...to smear on the belly and...to drink.”
	Pregnancy management rituals	Practices like “tying” the pregnancy or treating local conditions like “Nabuguma”	“You calculate...then the months pass...you say I will go to Mrs. Bizibu.”
	Spiritual or symbolic interpretations	Conditions like fetal positioning interpreted traditionally	“She touches the belly...tells you the baby is lying transversely.”
Health Risks and Limits of TBAs	Complex or emergency cases	TBAs refer to friends or other providers when complications arise	“She called her friend...a health worker...put the cannula.”
	Informal referral systems	Reliance on personal networks for emergency care	“She said she had a friend...called her and she came with a drip.”
	Lack of formal procedures (e.g., resuscitation)	Inadequate emergency newborn care skills	“She didnť know what to do...called a friend to help.”
Community Influence	Peer influence and word of mouth	Decision to go to a TBA based on community referrals	“The women on the village...told me that they had gone to the government hospitals but they weren’t helping.”
	Trust in TBAs	TBAs are respected and preferred in the community	“They like them very much...because of their ways, they care about the patients.”
Husband/Family Involvement	Spouse support and decision-making	Husband agreed with shift to TBA after bad hospital experience	“He accepted because of the condition of the first pregnancy.”

## Data Availability

The transcripts used and/or analyzed during the current study are available from the corresponding author upon reasonable request.
